# Lack of association of the *HMGA1* IVS5-13insC variant with type 2 diabetes in an ethnically diverse hypertensive case control cohort

**DOI:** 10.1186/1479-5876-11-12

**Published:** 2013-01-09

**Authors:** Jason H Karnes, Taimour Y Langaee, Caitrin W McDonough, Shin-Wen Chang, Miguel Ramos, James R Catlin Jr, Octavio E Casanova, Yan Gong, Carl J Pepine, Julie A Johnson, Rhonda M Cooper-DeHoff

**Affiliations:** 1 Division of Clinical Pharmacology, Vanderbilt University, 1275 Medical Research Building IV, Nashville, TN, 37232-0575, USA; 2 Department of Pharmacotherapy and Translational Research, University of Florida, HSC PO Box 100486, Gainesville, FL, 32610-0486, USA; 3 Division of Cardiovascular Medicine, University of Florida, PO Box 100277, Gainesville, FL, 32610-0486, USA

**Keywords:** HMGA1, Type 2 diabetes, Genetics

## Abstract

**Background:**

Recently, the high-mobility group A1 gene (*HMGA1*) variant IVS5-13insC has been associated with type 2 diabetes, but reported associations are inconsistent and data are lacking in Hispanic and African American populations. We sought to investigate the *HMGA1*-diabetes association and to characterize IVS5-13insC allele frequencies and linkage disequilibrium (LD) in 3,070 Caucasian, Hispanic, and African American patients from the INternational VErapamil SR-Trandolapril STudy (INVEST).

**Methods:**

INVEST was a randomized, multicenter trial comparing two antihypertensive treatment strategies in an ethnically diverse cohort of hypertensive, coronary artery disease patients. Controls, who were diabetes-free throughout the study, and type 2 diabetes cases, either prevalent or incident, were genotyped for IVS5-13insC using Taqman®, confirmed with Pyrosequencing and Sanger sequencing. For LD analysis, genotyping for eight additional *HMGA1* single nucleotide polymorphisms (SNPs) was performed using the Illumina® HumanCVD BeadChip. We used logistic regression to test association of the *HMGA1* IVS5-13insC and diabetes, adjusted for age, gender, body mass index, and percentage European, African, and Native American ancestry.

**Results:**

We observed IVS5-13insC minor allele frequencies consistent with previous literature in Caucasians and African Americans (0.03 in cases and 0.04 in controls for both race/ethnic groups), and higher frequencies in Hispanics (0.07 in cases and 0.07 in controls). The IVS5-13insC was not associated with type 2 diabetes overall (odds ratio 0.98 [0.76-1.26], p=0.88) or in any race/ethnic group. Pairwise LD (r^2^) of IVS5-13insC and rs9394200, a SNP previously used as a tag SNP for IVS5-13insC, was low (r^2^=0.47 in Caucasians, r^2^=0.25 in Hispanics, and r^2^=0.06 in African Americans). Furthermore, *in silico* analysis suggested a lack of functional consequences for the IVS5-13insC variant.

**Conclusions:**

Our results suggest that IVS5-13insC is not a functional variant and not associated with type 2 diabetes in an ethnically diverse, hypertensive, coronary artery disease population. Larger, more adequately powered studies need to be performed to confirm our findings.

**Trial registration:**

clinicaltrials.gov (NCT00133692)

## Background

Type 2 diabetes constitutes a major and growing health problem worldwide and is predicted to afflict 490 million by 2030 [1]. Type 2 diabetes has strong genetic influences and many polymorphisms have now been reproducibly associated with type 2 diabetes [2,3]. However, genome wide association studies (GWAS) explain only 10-15% of heritability and have not consistently improved diabetes risk prediction [4]. Low frequency variation may account for much of the missing heritability in type 2 diabetes risk and may help translate genetic association study results into clinical type 2 diabetes risk prediction.

Recently, the low frequency insertion polymorphism IVS5-13insC (c.136-14_136-13insC) in the high-mobility group A1 gene (*HMGA1*), a transcriptional regulator of the insulin receptor gene (*INSR*), was identified and associated with type 2 diabetes [5]. Whereas two studies have observed a significant positive association between IVS5-13insC and type 2 diabetes in Caucasian and Chinese populations [5,6], another study in Caucasians observed no association [7]. Data are lacking for IVS5-13insC in populations with African and Hispanic descent, which have disproportionately high type 2 diabetes prevalence [8]. Conflicting results for an *HMGA1* association with type 2 diabetes and the lack of data in diverse race/ethnic groups make clinical translation of the *HMGA1* IVS5-13insC genotyping especially difficult.

Evidence for the functional impact of the *HMGA1* IVS5-13insC variant is also conflicting. One study observed that *HMGA1* and *INSR* expression was decreased in diabetic carriers of IVS5-13insC versus wild type diabetic and non-diabetic patients [5]. Additionally, *INSR* protein expression and insulin-binding capacity was restored in lymphoblasts obtained from diabetic IVS5-13insC carriers by *HMGA1* DNA transfection. Another study observed no effect of IVS5-13insC on *HMGA1* or *INSR* expression in adipose tissue of normoglycemic patients [7]. IVS5-13insC occurs at position −13 of *HMGA1* exon 6, but the direct mechanism of the variant’s effects on mRNA expression or amino acid sequence remains unclear.

We tested the association of *HMGA1* IVS5-13insC with type 2 diabetes in an ethnically diverse population from the INternational VErapamil SR-Trandolapril STudy (INVEST). INVEST compared CV outcomes and NOD in hypertensive coronary artery disease patients treated with two antihypertensive treatment strategies. We also determined minor allele frequencies (MAF) and linkage disequilibrium (LD) for *HMGA1* variants and tested the functional impact of IVS5-13insC *in silico*.

## Methods

### Study design and participants

INVEST compared CV outcomes and incident diabetes in hypertensive, coronary artery disease patients at least 50 years of age during randomized treatment with either an atenolol-based or a verapamil sustained release (SR)-based antihypertensive treatment strategy. The design, primary outcome, and NOD results have been previously published in detail [9-12]. Briefly, the verapamil SR strategy consisted of stepped therapy with verapamil SR, trandolapril add-on, dose titration, then HCTZ add-on treatment for BP control and end organ protection as necessary. The atenolol-based strategy consisted of atenolol, HCTZ add-on, dose titration, then trandolapril add-on treatment as necessary. The INVEST GENEtic Substudy (INVEST-GENES) collected DNA samples from 5,979 INVEST patients at 187 sites in the United States and Puerto Rico.

We conducted a nested case control study including cases with type 2 diabetes at baseline (prevalent diabetes) or type 2 diabetes that developed during a mean 2.8 years follow-up (incident diabetes). Type 2 diabetes was determined by patient report and by site investigators from a review of all available patient data, including use of diabetic medication and lab measures [11]. We identified age, gender, and race/ethnicity-matched controls who remained diabetes-free over a mean 2.8 years follow-up. Age matching was performed after stratification by decade and we attempted to match cases and controls in a 1:1 ratio. The institutional review boards of participating study centers approved the study protocol and all patients provided written informed consent for participation in INVEST and additional written informed consent for genetic studies. INVEST is registered at clinicaltrials.gov (NCT00133692).

### Genotyping

Genotyping for *HMGA1* IVS5-13insC was performed using TaqMan® (Applied Biosystems, Foster City, CA, USA) with PCR primers and probe for IVS5-13insC (PN4331349) purchased from Applied Biosystems. For IVS5-13insC genotyping quality control, 5% of samples were genotyped in duplicate on Taqman®. A total of 612 Taqman genotypes were confirmed using pyrosequencing (Biotage AB, Uppsala, Sweden), using the following PCR and sequencing primers respectively: forward-biotinylated-5′-GGGGTGGAAACAGGTGATG-3′, reverse-5′-CACTTCGCTGGGCTCCTT-3′, and reverse-5′-TTCTGTAAAGACAGAGG-3′. Sanger sequencing was used to genotype 58 samples that showed discrepancies between Taqman® and Pyrosequencing platforms.

Genotyping for eight additional *HMGA1* single nucleotide polymorphisms (SNPs) was performed using the HumanCVD BeadChip and Infinium II Assay (Illumina, San Diego, CA) on 1,489 INVEST patients to perform LD analyses. The HumanCVD BeadChip contains approximately 50,000 cosmopolitan tag SNPs for 2,100 CV and metabolic-related genes [13]. HumanCVD BeadChip data quality was ensured in PLINK using genotype and sample call rates, concordance rates for blind duplicates, gender confirmation, cryptic relatedness using pairwise identity-by-descent, and estimation of heterozygosity using the inbreeding coefficient F [14]. Individuals were excluded if call rates were below 90 percent and SNPs were excluded if call rates were below 95 percent. In addition, 87 ancestry informative markers were genotyped in 2,860 INVEST patients to estimate Caucasian, African, and Native American ancestry using STRUCTURE [15]. Race/ethnic groups were determined by patient self-report with interaction by the study investigator [16] and confirmed using principal components analysis generated from LD-pruned HumanCVD BeadChip data and ancestry informative markers.

### Statistical analysis

All statistical analyses were performed using SAS version 9.2 (SAS, Cary, NC). Differences in patient characteristics comparing cases and controls at baseline were determined using t-tests and chi square tests, as appropriate. Deviations from Hardy Weinberg Equilibrium were assessed using a chi square test. Multi-variable logistic regressions were performed overall and by race/ethnic group to determine odds ratios (ORs) and 95% confidence intervals (95%CIs) for type 2 diabetes in IVS5-13insC variant carriers versus non-variant carriers. Variables for adjustment included age, gender, and body mass index (BMI) in order to maintain consistency with previously published *HMGA1* analyses [5,7]. Percentage of Caucasian, African, and Native American ancestry, as estimated by ancestry informative markers, was included as a variable for adjustment in analyses where all race/ethnic groups were combined.

The combined race/ethnic group analysis was considered primary with alpha=0.05, since IVS5-13insC was presumed to be a functional SNP with similar consequences across race/ethnic groups, based on previously published reports [5,7]. Assuming a MAF of 0.05 and OR of 1.40, we had 84% power to detect an association between the IVS5-13insC variant and type 2 diabetes in a dominant model in the overall population. Assuming a MAF of 0.05 and OR of 1.40, we had 61% power in Hispanics, 44% power in whites, and 18% power in blacks to detect an association by race/ethnicity. Pairwise LD values (r^2^) and LD plots were generated by race/ethnic group using Haploview [17]. We predicted *in silico* functional consequences of the IVS5-13insC variant using SNPNexus [18] and ESEfinder 3.0 [19].

## Results

We identified 446 incident type 2 diabetes cases over a mean 2.8 years of follow-up and genotyped an additional 1329 prevalent type 2 diabetes cases in INVEST-GENES. At baseline, patients with incident or prevalent type 2 diabetes had higher BMI and lower diastolic BP *versus* age, race/ethnicity, and gender-matched controls (Table 1). Cases also had a higher prevalence of hypercholesterolemia, left ventricular hypertrophy, and congestive heart failure.

**Table 1:** Characteristics of type 2 diabetes cases and controls at baseline

Characteristic*	**Type 2 diabetes cases (n=1775)**	**Controls (n=1295)**	***p *value**^**†**^
Age (years)	65.8 (9.2)	65.7 (9.1)	0.93
Female, n (%)	1,014 (57)	731 (56)	0.71
BMI (kg/m^2^)	30.6 (5.6)	28.9 (5.4)	<0.0001
Race/ethnicity, n (%)			0.15
Caucasian	608 (34)	493 (38)	
Hispanic	937 (53)	642 (50)	
African American	216 (12)	153 (12)	
Blood pressure (mm Hg)			
Systolic	149 (19)	148 (18)	0.32
Diastolic	85 (11)	86 (10)	0.0008
Hypercholesterolemia, n (%)^‡^	1,017 (57)	673 (52)	0.003
History of LVH, n (%)	319 (18)	174 (13)	0.0007
History of CHF, n (%)**	84 (5)	25 (2)	<0.0001
History of smoking, n (%)	706 (40)	507 (39)	0.73

BMI indicates body mass index; LVH, left ventricular hypertrophy; CHF, congestive heart failure.

*Values are mean ± standard deviation unless otherwise noted. †P values represent t-tests and chi square tests where appropriate. ‡History of or currently taking lipid-lowering medications. **New York Heart Association Class I-III.

The IVS5-13insC variant did not deviate from HWE in any race/ethnic group (Table 2). All *HMGA1* SNPs had call rates above 95 percent and no SNPs failed quality control based on call rate, Hardy Weinberg equilibrium testing, concordance with blind duplicates, or estimation of heterozygosity using the inbreeding coefficient F. The genomic inflation factor lambda was 1.03 for INVEST individuals genotyped for the *HMGA1* IVS5-13insC variant, suggesting minimal population stratification in genotyped individuals. For the IVS5-13insC variant, concordance of duplicates on Taqman® was 99% and concordance between Pyrosequencing and Taqman® was 95%. In Caucasians, the MAF of IVS5-13insC was 0.03 in diabetic cases and 0.04 in controls. The frequency of IVS5-13insC was similar in African American individuals (0.03 in cases and 0.04 in controls) and highest in Hispanics (0.07 in cases and 0.07 in controls). No significant associations were observed between the IVS5-13insC variant and diabetes overall (OR 0.98 95%CI 0.76-1.26, p=0.88). The IVS5-13insC variant was not associated with diabetes in any race/ethnic group (Table 2).

**Table 2:** IVS5-13insC genotype frequencies and associations with diabetes overall and by race/ethnicity

				**Unadjusted**		**Adjusted**	
**Race/ethnic group**	**MAF (cases)**	**MAF (controls)**	HWE p value*	**Odds ratio (95%CI)**	**p value**	**Odds ratio (95%CI)**^**†**^	**p value**^**†**^
Overall	0.053	0.052	-	1.00 (0.79-1.26)	0.98	0.98 (0.76-1.26)	0.88
(n=3070)							
Caucasian	0.028	0.036	0.40	0.75 (0.46-1.22)	0.25	0.95 (0.44-2.06)	0.90
(n=1101)							
Hispanic	0.074	0.070	0.20	1.09 (0.82-1.45)	0.57	0.79 (0.49-1.25)	0.31
(n=1579)							
African American (n=369)	0.030	0.039	0.10	0.83 (0.36-1.90)	0.65	1.51 (0.48-4.74)	0.48

95%CI indicates 95% confidence interval; MAF, minor allele frequency, HWE, Hardy Weinberg Equilibrium.

*Hardy Weinberg Equilibrium p value calculated using a chi square test by race/ethnicity in non-diabetic patients. †Generated using logistic regression in a dominant model adjusted for age, gender, body mass index, and Caucasian, African, and Native American ancestry as estimated by ancestry informative markers.

**Figure 1:**
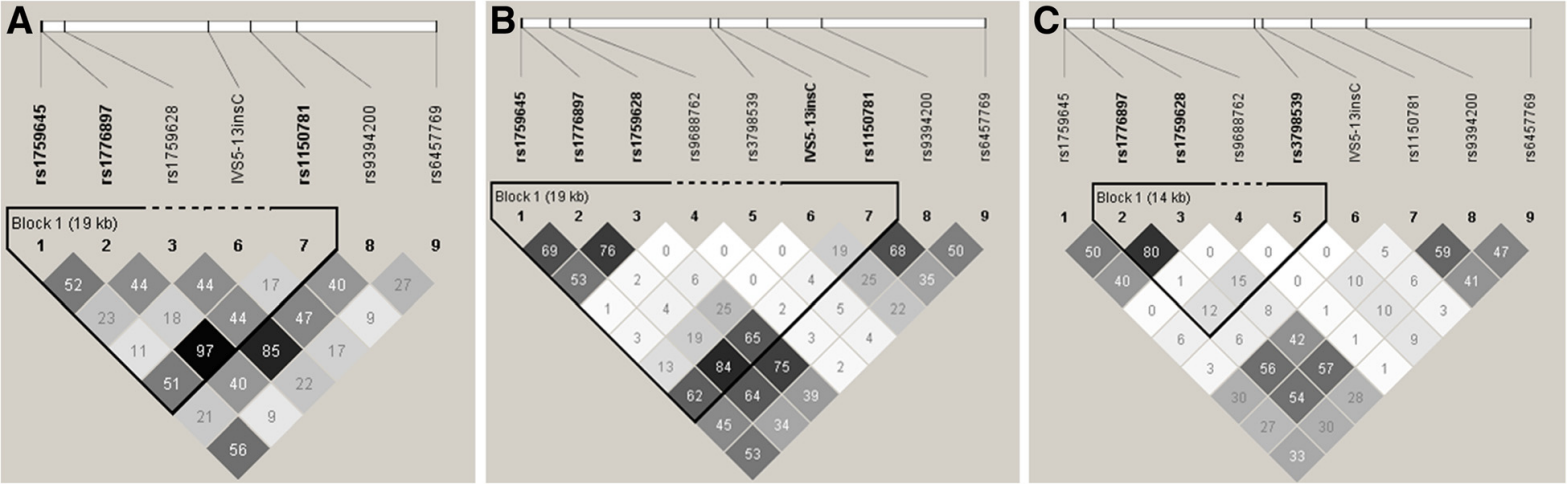
**Haploview-generated linkage disequilibrium (LD) plot of *HMGA1 *variation in INVEST Caucasians (A), Hispanics (B), and African Americans (C).** Regions of higher LD are shaded darker according to higher r^2^ values and pairwise r^2^ values are indicated in each box.

Pairwise LD of *HMGA1* SNPs by race/ethnicity is presented in Figure 1. Pairwise LD (r^2^) of IVS5-13insC and rs9394200, the SNP previously used as a tag SNP for IVS5-13insC [7], was 0.47 in Caucasians, 0.25 in Hispanics, 0.06 in African Americans. *In silico* functional analysis of IVS5-13insC revealed no predicted changes to amino acid sequence, conserved transcription factor binding sites, CpG islands, or miRNA regulatory sites. IVS5-13insC was not predicted to cause the creation or disruption of splice sites. However, *in silico* evaluation of exonic splice enhancer (ESE) sites revealed that the C insertion of IVS5-13insC creates an additional ESE motif for SF2/ASF (IgM-BRCA1).

## Discussion

In the present study, we observed that the *HMGA1* IVS5-13insC variant was not associated with diabetes in the INVEST-GENES population overall or in any race/ethnic group. We observed a frequency of IVS5-13insC consistent with previously published studies in Caucasians [5,7] and observed an increased frequency of this variant in Hispanics. Furthermore, pairwise LD in our population suggests that IVS5-13insC is not effectively tagged by rs9394200 in any race/ethnic group and *in silico* analysis revealed minimal evidence of putative functional consequences of the IVS5-13insC variant.

Our observations extend existing knowledge of genetic determinants of type 2 diabetes and are consistent with the findings of Marquez et al., which suggest a lack of association between IVS5-13insC and type 2 diabetes [7]. Marquez et al. also found that the IVS5-13insC variant is not functional, which is supported by our lack of observed association in multiple race/ethnic groups and our *in silico* functional results which predicted no important consequences from this variability*.* Our findings are inconsistent with two studies that observed a significant association between IVS5-13insC and type 2 diabetes [5,6]. This discrepancy may be due to differences in race/ethnicity of our population compared with other studies. However, a functional IVS5-13insC variant would be expected to have similar effects across multiple race/ethnicity groups.

Although our association analysis is consistent with the findings of Marquez et al. [7], our observation of low LD between IVS5-13insC and rs9394200 in all race/ethnic groups suggests that rs9394200 is not an appropriate tag SNP for IVS5-13insC. In Hapmap, the MAF of the T allele for rs9394200 is similar to the MAF for IVS5-13insC in Caucasians (0.03), but is 0.45 in Yorubans, further suggesting that rs9394200 does not adequately tag IVS5-13insC, especially in non-Caucasian populations. Furthermore, rs9394200 is 5000 base pairs downstream from the of *HMGA1* 3′ end and is represented on arrays utilized by type 2 diabetes GWAS [3,20]. Therefore, if rs9394200 were a significant contributor to risk for diabetes, it likely would have been identified in the GWAS analyses.

Our observation of a lack of association in Hispanics and African Americans suggests that IVS5-13insC may be associated with diabetes only in Caucasian individuals. While we did not observe even a trend towards association in our Caucasian population, we acknowledge that our power to observe an association in Caucasians is lower than in the previously published studies. Although we had 84 percent power to detect an association in our overall population, we had inadequate power to definitively conclude an association in by race/ethnicity analyses.

Our *in silico* analysis did not reveal any direct mechanism of the IVS5-13insC variant’s effects on mRNA expression or amino acid sequence, suggesting that the variant is not likely to be functional. Although the results of Chiefari et al. suggested a functional effect of the IVS5-13insC variant on *HMGA1* and *INSR* mRNA and protein expression in monocytes [5], Marquez et al. found no effect of the variant on mRNA expression in adipose tissue. The differences in observed effect on mRNA expression may potentially be explained by differential effects of the variant on transcription in monocytes and adipose tissue. The apparent functional effect of IVS5-13insC observed by Chiefari et al. may also be confounded by the lack of evaluation of IVS5-13insC variant carriers versus non-carriers among non-diabetic controls [7] or potential treatment with glucose-lowering medications in diabetic patients from whom monocytes were collected [21]. Finally, IVS5-13insC may have an unknown functional mechanism not identified by *in silico* tools used in this study. Our study has several limitations worthy of mention. We recognize the potential for false negative results in our analyses, especially in African Americans, considering the low frequency of the variant and the limited power to detect associations within each race/ethic group. Our observations require replication in independent populations of similar race/ethnic makeup. In INVEST, incident and prevalent diabetes diagnosis was based on investigator reports, but the diabetes phenotype is well described in a previous publication [11], the accuracy of such reporting has been verified by others [22], and has been used in other trials [23-25]. INVEST investigator-reported diabetes phenotypes have also been used in a large scale gene-centric meta-analysis with HumanCVD BeadChip data [26], suggesting validity with regard to the diabetes phenotype and genetic association analysis. Although our *HMGA1* association analysis may be confounded if control patients eventually develop diabetes after study follow-up, the high mean age and lower mean BMI suggests that our control patients are less likely to develop type 2 diabetes. In addition, although concordance within the Taqman® genotyping platform was high, our concordance between Taqman® and Pyrosequencing was 95%, suggesting some disagreement between platforms. However, Hardy Weinberg tests did not indicate genotype error and genotype error was minimized by utilization of Sanger sequencing for discrepancy confirmation.

## Conclusions

Our results suggest that the *HMGA1* IVS5-13insC is not associated with type 2 diabetes and may not have an important functional role in diabetes pathogenesis. We also provide frequency and LD data for Hispanic and African American populations, which have higher prevalence of type 2 diabetes. Our results also suggest that rs9394200 is not an effective tag SNP for *HMGA1* IVS5-13insC, especially in non-Caucasian populations. Functional studies and replication of these associations are needed to better define the potential role of *HMGA1* variants in predicting type 2 diabetes development. Although the current study suggests the lack of a functional role for IVS5-13insC, further study of *HMGA1* is warranted to clarify the role of this gene in diabetes pathogenesis. Larger, more adequately powered studies need to be performed to confirm our findings.

## Competing interests

The authors declare that they have no competing interests.

## Authors’ contributions

JHK drafted the manuscript and performed statistical analysis. TYL, JRC, MR, OEC, and SWC performed genotyping and helped draft the manuscript. CWM and YG performed HumanCVD BeadChip quality control procedures, contributed to statistical analysis and helped draft the manuscript. CJP, JAJ, and RCD conceived of the study, participated in its design and coordination, and helped draft the manuscript. All authors read and approved the final manuscript.
